# Laparoscopic or Open Adrenalectomy for Stage I–II Adrenocortical Carcinoma: A Retrospective Study

**DOI:** 10.3390/jcm12113698

**Published:** 2023-05-26

**Authors:** Martin Gaillard, Meva Razafinimanana, Alexandre Challine, Raphael L. C. Araujo, Rossella Libé, Mathilde Sibony, Maxime Barat, Jérôme Bertherat, Bertrand Dousset, David Fuks, Sebastien Gaujoux

**Affiliations:** 1 Department of Digestive, Hepatobiliary and Endocrine Surgery, Hôpital Cochin, APHP.Centre, 75014 Paris, France; mevraza@gmail.com (M.R.); bertrand.dousset@aphp.fr (B.D.); david.fuks@aphp.fr (D.F.); 2 Department of Digestive Surgery, Hôpital Saint-Antoine, APHP.Sorbonne Université, 75012 Paris, France; alexander.challine@aphp.fr; 3 Department of Surgery, Hospital Israelita Albert Einstein, Universidade Federal de Sao Paulo, Sao Paulo 05652-900, Brazil; raphael.araujo@unifesp.br; 4 Department of Endocrinology, Hôpital Cochin, APHP.Centre, 75014 Paris, France; rosella.libe@aphp.fr (R.L.); jerome.bertherat@aphp.fr (J.B.); 5 Department of Pathology, Hôpital Cochin, APHP.Centre, 75014 Paris, France; mathilde.sibony@aphp.fr; 6 Department of Radiology, Hôpital Cochin, APHP.Centre, 75014 Paris, France; maxime.barat@aphp.fr; 7 Department of Hepato-Biliary and Pancreatic Surgery and Liver Transplantation, Hôpital Pitié-Salpêtrière, APHP.Sorbonne Université, 75013 Paris, France; sebastien.gaujoux@aphp.fr

**Keywords:** adrenocortical carcinoma, adrenalectomy, laparoscopy

## Abstract

Surgical resection of adrenocortical carcinoma (ACC) is the only curative treatment. Even in localized (I–II) stages, open adrenalectomy (OA) is the gold standard, though laparoscopic adrenalectomy (LA) can be proposed in selected patients. Despite the postoperative benefits of LA, its role in the surgical management of patients with ACC remains controversial regarding oncologic outcomes. The aim of this retrospective study was to compare the outcomes of patients with localized ACC submitted to LA or OA in a referral center from 1995 to 2020. Among 180 consecutive patients operated on for ACC, 49 presented with localized ACC (19 LA and 30 OA). Baseline characteristics were similar between groups, except for tumor size. Kaplan-Meier estimates of 5-year overall survival were similar in both groups (*p* = 0.166) but 3-year disease-free survival was in favor of OA (*p* = 0.020). Though LA could be proposed in highly selected patients, OA should still be considered the standard approach in patients with known or suspected localized ACC.

## 1. Introduction

Adrenocortical carcinoma (ACC) is a rare and aggressive malignant tumor. Annual incidence is estimated between 0.5 and 2 cases per million inhabitants [1,2]. Most patients are diagnosed at an advanced stage, with large primary tumors invading adjacent organs or even metastatic spread. However, the widespread use of cross-sectional imaging has increasingly led to the discovery of ACC presenting as adrenal incidentalomas, nowadays estimated to be 20–30% of patients diagnosed with ACC [3]. Incidental diagnosis of ACC can be regarded as a chance for the patient because of the discovery at an earlier stage with the possibility of a complete surgical resection, which is the only curative treatment.

Laparoscopic adrenalectomy (LA) was initially described in 1992 [4] and became the gold standard for the surgical treatment of benign adrenal tumors [5] because of lower postoperative morbidity, shorter hospital stay, and shorter overall costs [6,7]. With increased surgical experience, LA was proposed for larger and malignant adrenal tumors. However, despite the benefits of LA, its role in the surgical management of patients with ACC remains controversial, particularly regarding oncologic outcomes, with reported increased risks of tumor capsule violation, tumor fragmentation, port-site or peritoneal carcinomatosis, and incomplete resection when compared to open adrenalectomy (OA) [8,9,10,11,12,13,14]. However, this remains controversial since other studies have shown comparable results between laparoscopic and open approaches [15,16,17,18,19,20,21].

The aim of this study was to report the surgical and oncologic outcomes of LA and OA in patients diagnosed with ENSAT stage I or II ACC in a single referral center.

## 2. Materials and Methods

### 2.1. Study Design

Between January 1995 and October 2020, data from all consecutive patients that underwent adrenalectomy for ACC in our surgical department were prospectively collected and retrospectively analyzed after institutional review board approval. Our center is the co-coordinating center of a nationally accredited network for adrenal cancers. Experience with LA procedures started in 1992 [22], and approximately 60 LA are performed annually. All adult patients that underwent adrenalectomy for primary ACC were included in the analysis. Diagnosis of ACC was afterward defined by pathological analysis with a Weiss score superior to 3. Tumors were classified according to the European Network for the Study of Adrenal Tumors (ENSAT) guidelines [23]. Patients with loco-regional (ENSAT stage III) or metastatic disease (ENSAT stage IV), as well as patients with oncocytic ACC, were not included in the study. Variables were compared in two groups of patients according to the surgical approach (LA or OA). Patients who underwent LA converted to OA were included in the LA group for intention-to-treat analysis. Inclusion and exclusion criteria are shown in Table 1.

### 2.2. Data Collection

Demographic data were obtained from a prospective database with additional retrospective medical records reviewed when necessary. Data recorded included patients’ and tumors’ characteristics, perioperative course, and follow-up details. Morbidity included every post-operative complication until discharge or included early readmission (less than 30 days). It was assessed with Clavien–Dindo classification [24].

### 2.3. Perioperative Management

Pre-operative assessment of adrenal mass included patient history, clinical examination, hormonal work-up (to identify autonomous excess of glucocorticoids, mineralocorticoids, sex hormones, adrenocortical steroid hormone precursors, and metanephrines), dedicated adrenal computed tomography (CT) without and with contrast injection, and whenever required magnetic resonance imaging (MRI) and ^18^F-fluorodeoxyglucose positron emission tomography combined with CT (FDG-PET/CT) as previously reported by our group [25]. All patients were discussed in a multidisciplinary team meeting (including the following disciplines: endocrinology, pathology, radiology, oncology, surgery, biochemistry, and nuclear medicine) expert in the field of adrenal pathology.

Critical parameters to strongly suspect ACC were young age (e.g., <40 years), rapidly developing symptoms, combined adrenocortical hormone excess or oversecretion of sex hormones or steroid hormone precursors, lesions >6 cm or showing rapid enlargement, lack of argument for a benign lesion on radiological imaging (i.e., CT density >10 Hounsfield units, characteristics of contrast washout values and chemical shift imaging) and increased uptake on functional imaging.

The decision of a laparoscopic or open approach was taken after a multidisciplinary discussion considering the parameters described above. In an attempt to clarify the choice of either the laparoscopic or open approach, we reviewed multidisciplinary meeting reports and operative reports in all patients to determine if adrenal masses were preoperatively considered to carry a high likelihood of malignancy.

The transabdominal approach in lateral decubitus was the standard approach for LA [26] with the adrenal tumor entirely removed in a bag with the peri adrenal tissue in order to prevent tumoral rupture. When the conversion was needed, a sub-costal incision with a possible sub-umbilical median extension was performed. Lymph node dissection and drainage were left to the surgeon’s evaluation.

Patients’ follow-up was assessed every 3 months during the first two years, then every 6 months, and finally every year. It consisted of a clinical evaluation, a thorough biological and adrenal hormonal investigation, and a radiological examination using thoracic, abdominal, and pelvic CT-scan, and FDG-PET/CT scan when necessary.

### 2.4. Statistical Analysis

Quantitative variables were expressed as mean ± standard deviation (SD) or median and interquartile range (IQR) and were compared using the Student *t*-test or the Mann–Whitney test, as appropriate. Qualitative variables were expressed as frequencies (percentages) and were compared using the Chi2 test or Fisher exact test, as appropriate. Disease-free survival (DFS) was defined as the time interval between the date of surgery and the date of recurrence diagnosis. Overall survival (OS) was defined as the time interval between the date of surgery and the date of death or last follow-up assessment for a live patient. Disease-free and overall survivals were estimated from Kaplan–Meier methods and survival curves were compared with the Log Rank test. Multivariate Cox regression analysis was carried out to evaluate the prognostic factors used for allocation to OA or LA, including age < 40 years, combined adrenocortical hormone excess or oversecretion of sex hormones or steroid hormone precursors, and tumor size > 6 cm.

Every test was bilateral, and a *p*-value inferior to 0.05 was considered statistically significant. Statistical analysis was carried out with R software (R Foundation for Statistical Computing, Vienna, Austria).

## 3. Results

### 3.1. Patients’ Characteristics

During the study period, 180 patients underwent surgery for ACC. Among them, 49 (27.2%) patients were operated on for stage I–II ACC, with a median age at surgery of 45 [33.0–60.8] years. There were 75.5% (*n* = 37) women. In these early stage ACC, most patients were diagnosed after screening for incidentaloma (40.8%). At first presentation, 38.8% (*n* = 19) of patients presented with endocrine disorders, which was Cushing’s syndrome in 35.0% (*n* = 17) of cases. Nineteen (38.8%) patients were operated on through a laparoscopic approach and were included in the LA group.

Patients in the OA group had lower body mass index (23.1 [20.2–25.6] kg/m^2^ versus 26.9 [23.2–30.9] kg/m^2^; *p* = 0.016) and larger tumor size (median size: 70 [55.0–82.5] versus 54 [43.5–61.0] mm; *p* = 0.010) when compared to patients in the LA group. Characteristics of patients before surgery are summarized in Table 2.

For patients in the OA group, all reports (100%, *n* = 30) considered the adrenal lesion to carry a high likelihood of malignancy. In the LA group, adrenalectomy was performed for indeterminate adrenal lesions in 15 cases and for adrenal lesions considered to carry a high likelihood of malignancy in 4 cases (21%). Adrenal lesions considered to carry a high likelihood of malignancy were more frequent in the OA group (*p* = 0.026). On multivariate Cox regression analysis, age < 40 years was an independent prognostic risk factor (*p* = 0.009). Repartition of patients with age < 40 years did not differ between groups (*p* = 0.57).

### 3.2. Intra and Postoperative Outcomes

The median duration of surgery was 120 (108–150) minutes and was significantly lower in the LA group (120 (100–120) versus 150 (135–175), *p* = 0.02) when compared to the OA group. Among the 19 patients in the LA group, 1 conversion occurred due to the high volume of the tumor i.e., 8 cm. Lymph node resection, defined as an effort of the surgeon to perform a lymph node resection according to the surgical protocol, was performed in 27 (57%) patients in the overall cohort, in 42.1% (*n* = 8) of patients in the LA group and in 63.3% (*n* = 19) of patients in the OA group (*p* = 0.25). No metastatic lymph node was found in either group. Intraoperative tumor capsule rupture occurred in 4 (8.2%) patients in the overall cohort, in 3 (15.8%) patients in the LA group, and in 1 (3.3%) patient in the OA group (*p* = 0.28). Postoperative severe complications (Clavien–Dindo score ≥ 3) occurred in 3 (6%) patients. Postoperative adrenal insufficiency occurred in 29 (59%) patients. No difference between groups was observed.

### 3.3. Pathologic Examination

In the overall cohort, 42 patients (85.7%) were classified as ENSAT stage II. Tumor size on pathological examination was larger in the OA group (77 mm (61–90) versus 62 mm (52–72), *p* = 0.03) when compared to the LA group. No difference between groups was observed regarding ENSAT stage, Weiss score, Ki67 proliferation index, mitotic index, microscopic rupture of tumor capsule, and R status. Characteristics of pathologic examination are presented in Table 3.

### 3.4. Survival Analysis

Overall, 25 (51.0%) patients with stage II disease received adjuvant mitotane, 10 (52.6%) patients in the LA group, and 15 (50.0%) patients in the OA group (*p* = 1.0). No adjuvant radiotherapy was performed. At the time of the analysis, recurrence occurred in 10 (20.4%) patients in the overall cohort, 8 (42.1%) patients in the LA group, and 5 (16.7%) patients in the OA group. Of the eight patients with recurrence in the LA group, isolated local recurrence was present in one (5.3%) patient and local recurrence associated with distant metastases was present in four patients (21.0%). No port site recurrence was observed. Of these eight patients with recurrence, four (21.0%) were offered surgical resection of one or more lesions. Of the five patients with recurrence in the OA group, isolated local recurrence was present in one (3.3%) patient and local recurrence with distant metastases was present in one (3.3%) patient. Of these five patients with recurrence, four (13.3%) were offered surgical resection of one or more lesions.

**Figure 1:**
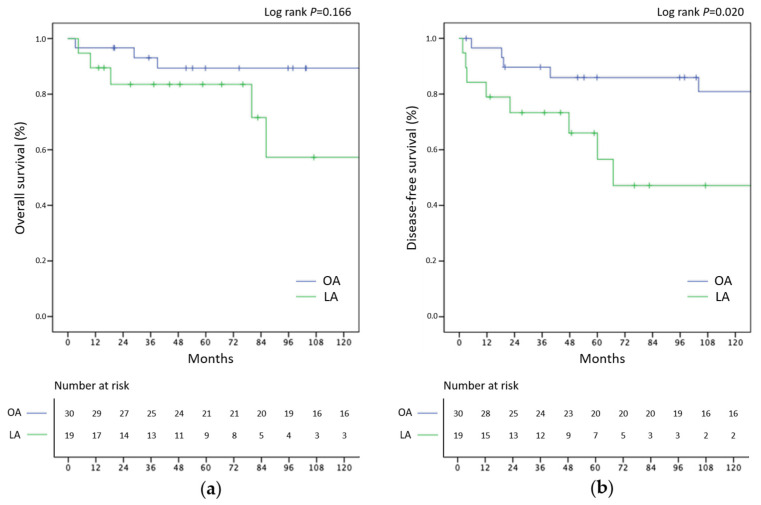
Overall survival (**a**) and disease-free survival (**b**) Kaplan–Meier curves between laparoscopic (LA) and open approaches (OA).

The 5-year overall survival for OA versus LA was 89.4% vs. 83.5% (*p* = 0.166). The median OS was not reached in both groups. The 3-year disease-free survival for OA versus LA was 89.7% vs. 73.3% (*p* = 0.020). The median DFS was 66.9 months in the LA group and was not reached in the OA group. The Kaplan–Meier curves of the OS and DFS are presented in Figure 1.

## 4. Discussion

Although there is consensus that surgery provides the best chance of cure for patients with ACC, it has to be underlined that “oncologic” resection of the adrenal tumor is a major prognostic factor [27], particularly regarding complete (R0) resection [28,29] and absence of tumor capsule rupture or spillage [30,31]. Since LA has demonstrated its unequivocal advantages over OA for the treatment of most adrenal masses (namely decreased blood loss, shorter recovery time, and reduced postoperative pain) [32,33], it seemed logical that expert centers would propose LA in the context of ACC. Such an approach can only be considered for localized (stage I–II) ACC, representing approximately 60% of all ACC [3], and cannot be proposed when suspecting stage III disease where OA is preferable [27,34]. The question of the appropriateness of LA for complete resection of localized ACC remains and is still a matter of debate [13]. As stage I–II tumors are confined to the adrenal capsule (without invasion of surrounding tissue, adjacent organs, lymph nodes, or distant metastases), at least from a theoretical point of view they are amenable to curative treatment by complete surgical resection including adrenal gland and peri adrenal fat. Nevertheless, due to the rarity of ACC, literature is scarce and more is needed. A randomized controlled trial comparing OA and LA in this specific context will likely never be performed, thus the question can only be answered through retrospective studies and meta-analysis, which have reported controversial outcomes and did not allow drawing clear conclusions [35,36].

In the present series, overall survival in patients treated with LA compared to OA did not show differences (*p* = 0.166), and differences in DFS were (3-year DFS was 66.7% for LA versus 80% for OA; *p* = 0.020). The previous series reported no statistically different oncological outcomes after LA versus OA for stage I–II ACC [16,19,20,21], all emerging from reference centers with stringent patient selection operated on by expert surgeons. Indeed, center volume and surgeon experience are of key importance to optimize the oncologic outcomes of patients with localized ACC [37]. However, three of these studies clearly excluded patients with incomplete (R1 or R2) resection [16,19,20]. Because no difference was found regarding oncologic outcomes, these reports concluded that LA is acceptable for localized ACC if the proper patient selection is achieved and oncological principles of surgery are respected. In our series of 49 patients, we reported 2 (4.1%) cases of R1 resection, both after LA. In another series focusing on LA versus OA in stage I–II ACC, Wu et al. did not observe any difference in terms of DFS or OS [21]. The rate of incomplete resection is not reported. However, the rates of local and peritoneal recurrence were higher after LA when compared to OA (42% vs. 22%, *p* = 0.035). In fact, the main drawback of LA is the risk of difficult exposure and dissection, which could lead to incomplete resection or intraoperative tumor rupture. In a series of 88 patients with stage I–III ACC, Miller et al. reported 50% incomplete resection or intraoperative tumor rupture during LA compared to 18% during OA (*p* = 0.01) [10]. The same group later reported longer OS (*p* = 0.002) and shorter time to local or peritoneal recurrence (*p* = 0.002) when focusing on patients with stage II ACC undergoing LA versus OA [11]. It should not be forgotten that LA can be converted to OA if necessary, and we believe surgeons should consider early conversion to an open approach when facing difficult exposure or dissection during LA for adrenal lesions considered to carry a high likelihood of malignancy or indeterminate. Additionally, a better preoperative selection of the cases than those exploited in this study would seem to be mandatory for the allocation of patients to OA or LA.

This study has several limitations that should be underlined. First, ours is a retrospective observational series that lends itself to a selection bias. In fact, even though the two groups were well-matched in terms of the main pathological findings, they differed for some baseline features, including BMI and tumor size which were lower and higher, respectively, in the OA group. Moreover, allocation to either surgical treatment was performed by a multidisciplinary team that apparently had no tools to assess preoperatively local spread, capsule invasion, technical difficulty of excision, etc. We also choose to include only patients with pathologically proven localized (stage I–II) ACC, which may have somewhat reduced the number of our cohort but considerably reduced the selection bias. Future studies focused on preoperative staging would also be helpful to assist surgeons in the choice of LA or OA procedures. As with other reports comparing LA versus OA in patients with ACC, our sample size is relatively small, and the statistical power of some analyses may be rather low due to the limited number of patients and events. Additionally, several factors may have impacted the results, such as adjuvant therapies, surgical volume, duration of follow-up, and others. Enrolled patients ranged widely, from 1985 to 2020, and equipment and surgical techniques have evolved, which may affect patient outcomes. Ideally, a meta-analysis of individual patient data should be performed, but it is time and resource-consuming, and access to necessary data over such a long period of time is unlikely.

Based on current literature, definitive conclusions regarding the appropriateness of LA for localized (stage I–II) ACC cannot be drawn because of the low quality of evidence from observational studies. Our findings strengthen the hypothesis that the oncologic effectiveness of LA in the treatment of ACC, even at an early stage, is to be considered with great prudence. Several guidelines have been published on the surgical management of ACC [27,34,38,39]. All these guidelines recommend that the management of patients with ACC should be limited to expert centers, and all claim for open approach as the standard of surgical care for confirmed or highly suspected ACC (including stage I–II). European guidelines accept LA as an option when dealing with a suspected malignant adrenal mass ≤ 6 cm without evidence of local or nodal invasion, though restricted to expert high-volume centers, by experienced surgeons, with respect to the principles of oncological surgery [27,34,38]. Conversely, American guidelines recommend OA in adrenal tumors with a high risk of being malignant because of the increased risk for local recurrence and peritoneal spread when performed laparoscopically [39,40].

## 5. Conclusions

The oncologic appropriateness of LA for localized (stage I–II) ACC is still a matter of debate. In patients with adrenal lesions considered to carry a high likelihood of malignancy, OA should still be considered the standard approach and LA should be reserved for highly selected patients and performed by surgeons with appropriate expertise.

## Figures and Tables

**Table 1:** Inclusion and exclusion criteria.

Inclusion Criteria	Exclusion Criteria
Age > 18	Stage III–IV ACC
ACC on pathological analysis (Weiss score > 3)	Oncocytic ACC
Adrenalectomy as a primary procedure	

ACC: adrenocortical carcinoma.

**Table 2:** Patients and tumor characteristics (*n* = 49).

Characteristics	Overall (*n* = 49)	LA (*n* = 19)	OA (*n* = 30)	*p*
Age (years), median [IQR]	45 [33.0–60.8]	45 [33.0–66.5]	43.1 [31.8–57.5]	0.61
Gender (female), n (%)	37 (75.5%)	16 (84.2)	21 (70.0)	0.43
ASA score > 2, n (%)	2 (4.1)	1 (5.3)	1 (3.3)	1.0
BMI (cm/kg^2^), median [IQR]	24.2 [21.6–27.3]	26.9 [23.2–30.9]	23.1 [20.2–25.6]	0.016
Diabetes, n (%)	2 (4.1)	1 (5.3)	1 (3.3)	1.00
Presentation				
Incidentaloma, n (%)	20 (40.8)	9 (47.4)	11 (36.7)	0.39
Tumoral symptoms, n (%)	10 (20.4)	2 (10.5)	8 (26.7)	
Endocrine disorders, n (%)	19 (38.8)	8 (42.1)	11 (36.7)	
Tumor size (mm), median [IQR]	60 [47.8–78.8]	54 [43.5–61.0]	70 [55.0–82.5]	0.010
Side (left), n (%)	26 (53.1)	13 (68.4)	13 (43.3)	0.15

LA: laparoscopic adrenalectomy; OA: open adrenalectomy; IQR: interquartile range; ASA: American Society of Anesthesiologists; BMI: body mass index.

**Table 3:** Pathologic characteristics of the overall population.

Characteristics	Overall (*n* = 49)	LA (*n* = 19)	OA (*n* = 30)	*p*
ENSAT classification				
Stage I, n (%)	7 (14.3)	3 (15.8)	4 (13.3)	
Stage II, n (%)	42 (85.7)	16 (84.2)	26 (86.7)	1.00
Tumor size (mm), median (IQR)	70 (55–85)	62 (52.0–72.5)	77.5 (61.2–90.0)	0.03
Weiss score, median (IQR)	6 (4–7)	5 (3.5–6.5)	6 (4.0–6.8)	0.70
Ki67 index (%), median (IQR)	6 (3–15)	9.5 (3.5–22.5)	5 (3.0–10.2)	0.36
Unknown, n (%)	24 (49.0)	5 (26.3)	19 (63.3)	
Mitotic index, median (IQR)	6 (4–15)	6 (3.8–9.8)	5 (5.0–17.5)	0.75
Unknown, n (%)	10 (20.4)	3 (15.8)	7 (23.3)	
Microscopic rupture of tumor capsule, n (%)	8 (16.3)	4 (21.1)	4 (13.3)	0.75
R status				
R0	47 (95.9)	17 (89.5)	30 (100.0)	
R1	2 (4.1)	2 (10.5)	0 (0.0)	0.28

LA: laparoscopic adrenalectomy; OA: open adrenalectomy; IQR: interquartile range.

## Data Availability

The data presented in this study are available on request from the corresponding author. The data are not publicly available due to privacy reasons.
